# Establishing links between drug registers and essential medicines lists

**DOI:** 10.2471/BLT.24.291512

**Published:** 2024-10-29

**Authors:** Petra Brhlikova, Zaheer-Ud-Din Babar, Allyson M Pollock

**Affiliations:** a Population Health Sciences Institute, Newcastle University, Baddiley-Clark Building, Newcastle upon Tyne NE2 4AX, England.; b College of Pharmacy, Qatar University, Doha, Qatar.

## Abstract

Access to essential medicines is still suboptimal in many countries. Recent studies examining the registration of medicines at the country level show that a considerable proportion of essential medicines do not have any corresponding products registered for use at the country level and therefore cannot be available at all times. Conversely, many non-essential medicines are registered by regulatory authorities for local markets, potentially facilitating inappropriate drug use and antimicrobial resistance. Addressing this public health gap requires linking the data on national drug registers with national essential medicines lists. Achieving this linkage will necessitate the development of common data variables and standards for both drug registers and essential medicines lists. This linkage would provide medicines regulators and health policy-makers with information on the gaps in the registration of essential medicines and allow them to take steps to prioritize registration of these medicines. This approach would improve availability of essential medicines for public health priorities and prevent over-registration of unnecessary medicines.

## Résumé

L'accès aux médicaments essentiels demeure insuffisant dans de nombreux pays. Des études récentes consacrées à l'enregistrement des médicaments à l'échelle du pays montrent qu'il n'existe aucun produit équivalent homologué sur le territoire pour la plupart de ces médicaments, qui ne sont par conséquent pas disponibles à tout moment. À l'inverse, de nombreux médicaments non essentiels sont homologués par les autorités de réglementation pour les marchés locaux, ce qui pourrait favoriser une utilisation inadéquate et une résistance aux antimicrobiens. Remédier à ce problème de santé publique implique la création de liens entre les données figurant dans les registres nationaux de médicaments et les listes nationales de médicaments essentiels. Pour établir ces liens, il faudra développer des normes et variables de données communes aux registres et aux listes. Les organismes de réglementation compétents ainsi que les responsables de la santé pourraient ainsi obtenir des informations concernant les médicaments essentiels n'ayant pas encore été approuvés, leur permettant de prendre des mesures visant à les privilégier. Une telle approche améliorerait la disponibilité des médicaments essentiels pour les priorités de santé publique, et éviterait l'enregistrement abusif de médicaments superflus.

## Resumen

El acceso a los medicamentos esenciales sigue siendo deficiente en muchos países. Estudios recientes que examinan el registro de medicamentos a nivel nacional muestran que un porcentaje considerable de los medicamentos esenciales no tienen ningún producto correspondiente registrado para su uso a nivel nacional y, por lo tanto, no pueden estar disponibles en todo momento. Por otra parte, las autoridades reguladoras registran muchos medicamentos no esenciales para los mercados locales, lo que puede facilitar el uso inadecuado de fármacos y la resistencia a los antimicrobianos. Para subsanar esta deficiencia de la sanidad pública es preciso vincular los datos de los registros nacionales de medicamentos con las listas nacionales de medicamentos esenciales. Lograr esta vinculación exigirá el desarrollo de variables de datos y estándares comunes tanto para los registros de medicamentos como para las listas de medicamentos esenciales. Esta vinculación proporcionaría a los reguladores de medicamentos y a los responsables de elaborar las políticas sanitarias información sobre las deficiencias en el registro de medicamentos esenciales y les permitiría tomar medidas para dar prioridad al registro de estos medicamentos. Este planteamiento mejoraría la disponibilidad de medicamentos esenciales para las prioridades de salud pública y evitaría el registro excesivo de medicamentos innecesarios.

## ملخص

لا يزال الوصول إلى الأدوية الأساسية دون المستوى الأمثل في العديد من الدول. وتشير الدراسات الحديثة التي تفحص تسجيل الأدوية على مستوى الدولة، أن نسبة كبيرة من الأدوية الأساسية ليس لها أي منتجات مقابلة مسجلة للاستخدام على مستوى الدولة، وبالتالي لا يمكن أن تكون متوفرة في جميع الأوقات. وعلى العكس من ذلك، فإن العديد من الأدوية غير الأساسية يتم تسجيلها من جانب السلطات التنظيمية للأسواق المحلية، مما قد يسهل الاستخدام غير المناسب للأدوية ومقاومة مضادات الميكروبات. إن معالجة هذه الفجوة في الصحة العامة تتطلب ربط البيانات الموجودة في سجلات الأدوية الوطنية بقوائم الأدوية الأساسية الوطنية. وسوف يتطلب تحقيق هذا الربط تطوير متغيرات ومعايير بيانات مشتركة لكل من سجلات الأدوية وقوائم الأدوية الأساسية. ومن شأن هذا الربط أن يوفر للجهات التنظيمية للأدوية وواضعي السياسات الصحية، معلومات عن الفجوات في تسجيل الأدوية الأساسية، ويسمح لهم باتخاذ خطوات لإعطاء الأولوية لتسجيل هذه الأدوية. ومن شأن هذا الأسلوب أن يحسن من توفر الأدوية الأساسية لأولويات الصحة العامة، ويحول دون الإفراط في تسجيل الأدوية غير الضرورية.

## 摘要

在许多国家，获取基本药物的情况仍然不够理想。最近对国家层面药品登记情况的研究表明，相当一部分基本药物在国家层面的可供使用产品中并没有任何相应登记 记录，因此无法随时提供。相反，监管机构为当地市场登记了许多非必需药物，这可能会导致用药不当的情况加剧和抗菌素耐药性提高。解决这一公共卫生差距问题需要将国家药品登记册的数据与国家基本药物清单关联起来。如要建立此类关联性，需要为药物登记册和基本药物清单制定通用的数据变量和标准。这种关联性有助于药品监管机构和卫生政策制定者及时了解基本药物登记差距相关信息，并确保他们能够采取措施以优先登记这些药物。这一做法有利于根据公共卫生优先级提高基本药物的可用性，并防止非必要药物的过度登记。

## Резюме

Во многих странах доступ к основным лекарственным средствам все еще остается неоптимальным. Результаты недавно проведенных исследований, посвященных регистрации лекарственных средств на уровне стран, свидетельствуют о том, что значительная часть основных лекарственных средств не имеет соответствующих препаратов, зарегистрированных для использования на уровне страны, поэтому не может быть доступна на постоянной основе. И наоборот, многие лекарства, не являющиеся жизненно необходимыми, регистрируются регуляторными органами для местных рынков, что потенциально способствует ненадлежащему использованию лекарств и развитию устойчивости к противомикробным препаратам. Для устранения этого пробела в здравоохранении необходимо связать данные национальных реестров лекарственных средств с национальными списками основных лекарственных средств. Для достижения такой взаимосвязи потребуется разработка общих переменных данных и стандартов для реестров лекарственных средств и списков основных лекарственных средств. Такая связь предоставила бы уполномоченным органам и лицам, ответственным за разработку политики в области здравоохранения, информацию о пробелах в регистрации основных лекарственных средств и позволила бы им принять меры по приоритетной регистрации этих лекарственных средств. Такой подход позволит повысить доступность основных лекарственных средств для решения приоритетных задач здравоохранения и предотвратить избыточную регистрацию ненужных лекарственных средств.

## Introduction

Access to essential medicines and vaccines is a key component of universal health coverage and central to sustainable development goal target 3.8. Essential medicines are intended to be available within a functioning health system at all times, in sufficient quantities, in the appropriate dosage forms, with assured quality and at prices affordable to individuals and the community.^1^ By 2017, 40 years after the World Health Organization (WHO) launched the first essential medicines list, 137 countries, including 21 high-income countries, had a national essential medicines list in place.^2^ Most countries use the list to prioritize medicines by public health needs and guide public procurement and reimbursement of medicines.^3^ A 2014 meta-analysis of WHO and Health Action International surveys in 23 low- and middle-income countries showed higher median availability of essential medicines over non-essential medicines; 61.5% and 27.3%, respectively.^4^

Nevertheless low- and middle-income countries struggle to ensure people can access the full set of essential medicines on national essential medicines lists for a range of reasons, such as budgetary constraints, supply-chain bottlenecks, stockouts and limited local manufacturing of medicines.^5^^,^^6^ Pricing is another well-known and important factor affecting access to and availability of medicines. Most low- and middle-income countries have their own policies for controlling medicine prices and regulate medicine prices to some degree.^7^ An under-recognized and under-researched issue that affects access to essential medicines before pricing is the registration of medicines for use and sale in countries. Registration means the national medicines authority authorizes medicines on the basis of their safety, efficacy and quality before they can enter the country market. Exceptionally, a product may not be registered when authorities have approved its import and use in emergency situations or on a restricted basis (for example, special import licences). WHO recommends that each essential medicine has at least three different products registered at the national level to ensure a reliable supply of medicines.^8^^,^^9^

The regulatory authorities often work in isolation from the country’s national essential medicines committees. Currently, the necessary systems to link product registrations with national essential medicines lists are lacking in all countries. As a result, there is a misalignment between public health need for medicines and their market registration. To improve the availability of essential medicines and promote their rational use, we propose that, as part of the registration process, regulatory authorities be required to consider whether the product is included in the essential medicines list.

## Misalignment of registration

Medicines regulatory authorities do not proactively solicit or select which products to consider for registration in the given country. Instead, manufacturers submit applications to national medicines regulatory authorities to be able to market and sell a particular medicinal product. Products put forward by manufacturers may or may not be listed in the country’s essential medicines list, and may or may not respond to national public health needs. Indeed, the decision of manufacturers to apply for market registration is largely based on market profitability not whether the product is a public health priority. Factors that may discourage manufacturers from applying for a licence include long assessment times, price controls, and low demand due to lack of government funding to buy essential medicines and unaffordable prices for patients paying out-of-pocket.^10^^–^^12^

As a result, some essential medicines have no corresponding products registered (under-registration), while others have multiple products registered. In an audit comparing the medicines authorized on national drug registers with those on the essential medicines lists in Kenya, Uganda and the United Republic of Tanzania, 27.7% (175/632), 40.1% (266/663) and 50.2% (400/797) of essential medicines, respectively, had no registered products in 2018.^13^ For instance, in 2018, a formulation of the essential anaesthetic halothane was registered in the United Republic of Tanzania but not in Kenya or Uganda. However, there were no registered products corresponding to the anaesthetic gas isoflurane, despite it being included in the essential medicines list in these countries. Further, oral rehydration salts, which are on the essential medicines list, were registered in Uganda, but not in Kenya or the United Republic of Tanzania. Therapeutic classes such as medicines for parkinsonism, antidotes and other substances used in poisoning were particularly affected by a lack of registered products across the three countries.^13^ Moreover, the audit showed that a substantial proportion of the medicines registered had only one or two registered products – 37.6% (91/242) in Kenya, 36.1% (84/233) in Uganda and 44.9% (93/207) in the United Republic of Tanzania.^13^ The registration of these medicines was lower than the WHO-recommended threshold of three registered products.

A document analysis of the 2018 essential medicines list and registration status of medicines in Pakistan^14^ showed that 25.9% (179/690) of essential medicines were not registered at the country level. Here too, medicines for parkinsonism (100.0%, 2/2) and antidotes and other substances used in poisoning (60.0%, 6/10) were missing registered products.

At the same time, the policies of national medicines authorities have resulted in an excess of non-essential medicines approved for the local market, potentially facilitating inappropriate drug use and antimicrobial resistance.^15^ In Kenya, 20.6% (33/160) of antimicrobials listed on national essential medicines lists had no registered products. In Uganda and the United Republic of Tanzania, this proportion was 26.7% (50/187) and 28.6% (52/182), respectively. In contrast, 64.3% (1353/2105) of the registered antimicrobials in Kenya were not on the essential medicines list. In Uganda and the United Republic of Tanzania, this proportion was 51.1% (798/1563) and 53.2% (706/1327), respectively.^16^ For example, a fixed-dose combination of ampicillin and cloxacillin is specifically not recommended by WHO because it “is not evidence-based, nor recommended in high-quality international guidelines.”^17^ Although this combination was not included in the national essential medicines list of Kenya or Uganda, more than 20 versions of this medicine were on their national drug registers. Diclofenac was not included in Kenya’s essential medicines list, but 85 versions were registered in the country.^13^

National medicines regulatory authorities can facilitate the registration of prespecified products that respond to public health priorities, such as those prequalified by WHO and assessed collaboratively at the regional level (for example, ZAZIBONA,^18^ a collaborative medicines registration in Southern Africa), or medicines in prespecified classes such as antituberculosis, antiretroviral or antimalarial medicines. These pathways can fill some access and availability gaps but do not explicitly prioritize all medicines listed in national essential medicines lists. For example, medicines for parkinsonism and antidotes mentioned previously would not be eligible for such facilitated registration pathways.

Misalignment of essential medicines lists with product registration and national drug registers therefore undermines access to essential medicines. Meanwhile, plenty of non-essential medicinal products with the potential to cause harm are available. For this reason, we call on WHO to focus attention on national drug registers to ensure that they can be easily linked to and analysed with essential medicine listings.

## Linking drug registers and essential medicines lists

### National drug registers

National drug registers contain information on medicinal products authorized by the national medicine regulatory authority in their respective country. Currently, drug registers do not record whether the product is on the country essential medicines list or not. Moreover, many low- and middle-income countries rely on paper-based processes^19^ which make drug registers difficult to access and analyse. Digitization of the systems would ensure greater transparency and data sharing with various stakeholders.

The regulatory authorities of Kenya, Uganda and the United Republic of Tanzania, for instance, make their national drug registers publicly available on their institutional websites, allowing the information to be exported.^20^^–^^22^ Drug registers of many countries, however, are not so easily accessible and the information provided might be limited and not standardized. For example, drug registers in Kenya, Uganda and the United Republic of Tanzania provide the international non-proprietary name (INN; that is, generic name which identifies the pharmaceutical substances or active pharmaceutical ingredients), dosage form and strength, as well as information on producers and their location (Box 1). In contrast, the drug regulatory authority of Pakistan provides a provisional online drug register only listing the INN and company name.^23^ In India, a list of authorized medicines (INN, dosage form and strength) is available from the central regulatory authority,^24^ but manufacturing licences for the authorized medicines are issued by the state regulators and are not publicly available.^25^ In high-income countries, the drug registers are searchable for individual products and INNs, however, they do not publish the complete list of authorized medicinal products.^26^^,^^27^

Box 1 **Selected variables reported in national drug registers and essential medicines lists of Kenya, Uganda and the United Republic of Tanzania**

***Kenya***

**National drug register**

INN; dosage form, strength; pharmacotherapeutic group; route of administration; ATC

code; brand name; local technical representative; market authorization holder; production site; and registration status

**Essential medicines list**

INN; dosage form, strength; therapeutic class; and health-system level

***Uganda***

**National drug register**

INN; dosage form, strength; brand name; local technical representative; licence holder; manufacturer and manufacturing country; and registration date

**Essential medicines list**

INN; dosage form, strength; therapeutic class; health-system level; and VEN classification

***United Republic of Tanzania***

**National drug register**

INN; dosage form, strength; brand name; local technical representative; registrant; manufacturer and manufacturing country; and registration status

**Essential medicines list**

INN; dosage form, strength; therapeutic class; and health-system level

ATC: anatomical therapeutic chemical; INN: international non-proprietary name; NR: not reported; VEN: vital, essential and non-essential.

### Essential medicines lists

National essential medicines lists usually follow the WHO model list and contain information on the INN, dosage form and strength of recommended medicines. In addition, they may include information on the level of the health system at which the listed medicine is to be available (Box 1). Although WHO recommends that the registration status of medicines be considered when revising country essential medicines lists, in practice it seems this process does not occur.^28^ Essential medicines lists are more easily found and typically downloadable in portable document format (pdf) from the website of health ministries or drug regulatory authorities. The data, however, cannot be easily extracted and compared with drug registers or other essential medicines lists.

### Linkage

Digital linking of the information on registered medicinal products with the essential medicines list would enable monitoring of the registration status of essential medicines. This monitoring would in turn provide useful evidence for regulators and health policy-makers to prioritize missing essential medicines, and thus improve their availability and promote their rational use. They may want to offer incentives for manufacturers to apply for a licence to market missing essential medicines.

The linkage requires drug registers to standardize the key variables in line with those on essential medicines lists. Maintaining the linkage should be the joint responsibility of the regulator and the essential medicines list committee. Box 1 lists and compares selected variables for the drug registers and essential medicines lists of Kenya, Uganda and the United Republic of Tanzania. The box shows that there are three variables in common (INN, dosage form and strength). These three variables provide a bridge for linking and analysing.

As a priority and to enable the routine monitoring of the proportion of essential and non-essential medicines in the drug register, drug registers must record the essential medicines list status of all registered products. The auditing of the proportion of essential and non-essential medicines in the drug register would show the focus of regulatory activities. A further examination of the subset of registered non-essential medicines would enable identification of over-registration of specific classes of non-essential medicines, for example, antimicrobials, where action is needed to ensure their appropriate use.

For the essential medicines lists, the additional variable of interest is the registration status. Routine monitoring of registration status of essential medicines would identify medicines that the drug regulators should prioritize for registration.

### Other benefits

Medicines policies are fragmented and the responsibility of many departments. Data linkage would encourage communication and collaboration between institutions and departments responsible for drug registration, essential medicines list, pricing, procurement, treatment guidelines and prescribing. Additionally, WHO supports local production of essential medicines as a strategy to improve access to medicines.^29^ The analysis of national drug registers and essential medicines lists would enable identification of locally produced essential medicines. Subject to technical capacities, this information could lead to the formulation of targeted incentives for local or regional manufacturers to focus on missing essential medicines that address local health needs.^30^

## Way forward

The *Lancet* commission on essential medicines for universal health coverage identified five key challenges to universal equitable access to essential medicines, namely: sufficient financing; affordability; assurance of quality and safety; appropriate use; and research into and development of missing essential medicines to address unmet needs.^5^ To this list of challenges we add a sixth: the extent to which essential medicines are registered for use within country on national drug registers. This information is a vital indicator of availability.^9^^,^^31^

A first step is the need to ensure that national drug registers are publicly available and accessible in a digitized and standardized format. Digitization is necessary because most national drug registers contain many thousands of medicinal products, making the manual task of identifying registered essential medicines almost impossible. Digitizing registers would require WHO to develop common data standards and a data dictionary outlining the content and meaning of variables to be included in drug registers.^32^ Such standards and terms would ensure that the data collected in registers and essential medicines lists can be compared at the country level and also allow cross-country comparisons. The minimum set of variables should include the INN, strength, pharmaceutical form, authorized manufacturer and country of production, as well as essential medicine status. The standardization could build on WHO’s drug medicinal information dictionary that underpins the global monitoring of safety issues by Uppsala Monitoring Centre^33^ and the MedMon tool developed by WHO for monitoring the availability and affordability of medicines.^34^ Common data standards are crucial for countries that are already moving towards developing and optimizing publicly available electronic national drug registers. Regional regulatory harmonization initiatives such as the one in countries of the East African Community^35^ could provide a useful platform for piloting.

A second step would be to ask countries to pass new rules requiring regulators to prioritize the registration of all medicines on a national essential medicines list. This step might need to be accompanied by additional incentives to attract manufacturers of essential medicines, such as priority review and different registration fees: lowest fees for medicines on the national essential medicines list and single generics; higher fees for non-essential medicines and combination products; and the highest fees for new chemical products not registered in countries with stringent regulatory authorities. As in the United States of America, regulators could be required to coordinate with other stakeholders, such as medicine procurers and (local) manufacturers, to ensure availability of essential medicines.^36^

### Limitations

Our proposal has some limitations. Many low- and middle-income countries do not have digitized drug registers yet. As countries begin to upgrade their regulatory systems to a digital format, it is essential they first review their drug registers, their timeliness, the information available and its format. To allow the data linkage, the digitization requires developing data dictionaries and standards on information recording and sharing between those responsible for essential medicines lists and drug registers. The data linkage needs to be followed by a commitment to prioritize registration of essential medicines. The design of incentives to attract manufacturers of essential medicines needs to be well thought through to avoid unintended consequences. In South Africa, for instance, policies in favour of generics overwhelmed the regulatory authority and slowed down dossier assessment for all products.^37^ All interventions and use of incentives would require careful evaluation. More evidence is also needed on effective generic competition, and the number of medicinal products (with a different source of active pharmaceutical ingredient) required to ensure affordable prices and sustainable supply.

While drug registers are updated on a continuous basis, national essential medicines lists are updated less frequently, every 2 or more years. With every revision of an essential medicines list, the essential medicine status of products listed in the drug register would need to be updated in line with the deletions and additions in the revised essential medicines list.

## Conclusion

A gap exists between essential medicines lists and marketing authorizations which affects public health. Understanding the gap and setting the priorities accordingly would enable medicines regulatory authorities to focus on essential medicines and regional and country strategies for local production. Combined with routine monitoring of registration status of essential medicines, this approach would strengthen the role of essential medicines within the public health system, improve availability of medicines for public health priorities, and prevent over-registration of unnecessary medicines and the many harms that can follow.
